# Effect of multi-dimensional sensory touch and family-accompanied whole-process nursing on feeding intervention for preterm infants

**DOI:** 10.1590/1980-220X-REEUSP-2025-0128en

**Published:** 2025-11-21

**Authors:** Ting Ye, Lijuan Gao

**Affiliations:** 1 Changzhou Maternal and Child Health Care Hospital, Changzhou, Jiangsu Province, China.

**Keywords:** Family, Diet, Nursing, Infant, Premature, Touch, Família, Dieta, Enfermagem, Recém-Nascido Prematuro, Tato

## Abstract

**Objective:**

To assess the effect of multi-dimensional sensory touch and family-accompanied whole-process nursing on feeding intervention for preterm infants.

**Methods:**

A retrospective experimental study was designed to recruit 100 preterm infants admitted and treated between January 2022 and January 2024 as the subjects, with an intervention group (n = 50, undergoing multi-dimensional sensory touch plus family-accompanied whole-process nursing treatment) and a control group (n = 50, receiving conventional nursing treatment) established according to different nursing protocols.

**Results:**

The intervention group had significantly shorter transition time to oral feeding, extubation time of gastric tube, and length of hospital stay than the control group (P < 0.05). The Neonatal Behavioral Neurological Assessment Scale (NBNA) score significantly increased in the intervention group compared with that in the control group (P < 0.05). At 1 d before discharge, a significant increase in the Apgar score was detected from the intervention group compared to that from the control group (P < 0.05).

**Conclusion:**

Multi-dimensional sensory touch and family-accompanied whole-process nursing can significantly improve the overall physical signs of preterm infants and gradually facilitate their development.

## RESUMO

**Objetivo:**

Nosso objetivo foi avaliar o efeito do toque sensorial multidimensional e da enfermagem de processo integral acompanhada pela família na intervenção de alimentação para bebês prematuros.

**Métodos:**

Um estudo experimental retrospectivo foi projetado para recrutar 100 bebês prematuros admitidos e tratados entre janeiro de 2022 e janeiro de 2024 como sujeitos, com um grupo de intervenção (n = 50, submetidos ao toque sensorial multidimensional na enfermagem como processo integral acompanhada pela família) e um grupo controle (n = 50, recebendo enfermagem convencional) estabelecidos de acordo com diferentes protocolos de enfermagem.

**Resultados:**

O grupo de intervenção teve tempo de transição para alimentação oral, tempo de extubação da sonda gástrica e tempo de internação hospitalar significativamente menores do que o grupo controle (P < 0,05). A pontuação da *Neonatal Behavioral Neurological Assessment Scale* (NBNA) aumentou significativamente no grupo de intervenção em comparação com a do grupo controle (P < 0,05). Um dia antes da alta, um aumento significativo no escore de Apgar foi detectado no grupo de intervenção em comparação com o do grupo controle (P < 0,05).

**Conclusão:**

O toque sensorial multidimensional e a amamentação completa acompanhada pela família podem melhorar significativamente os sinais físicos gerais de bebês prematuros e facilitar gradualmente seu desenvolvimento.

## INTRODUCTION

Preterm infants, born before reaching the term-equivalent age, typically exhibit immature development of various organs and systems. This immaturity results in notable differences in both appearance and physiological functions compared to full-term infants. In particular, underdevelopment of the respiratory, immune, and thermoregulatory systems makes them especially vulnerable to complications such as hypoxia, unstable body temperature, and increased risk of infection, thereby posing significant challenges for clinical care and nursing^(1,2,3)^. Furthermore, limited medical resources and high workloads in pediatric settings can compromise the timeliness and quality of conventional nursing care, negatively affecting the prognosis and survival of these vulnerable infants.

To address these challenges, multi-dimensional sensory touch has emerged as a therapeutic approach that engages multiple senses—including vision, hearing, touch, smell, and taste—to evoke integrated perceptual experiences and emotional responses at both physiological and psychological levels, thereby supporting infant development^(4,5)^. At the same time, family-accompanied whole-process nursing, a patient-centered care model that emphasizes continuous family involvement and comprehensive care, has been shown to improve comfort and stability in neonatal care^(6)^.

Recently, clinicians have begun integrating these two interventions into feeding strategies, offering both sensory stimulation and consistent familial support throughout the hospital stay. This combined approach aims to address the physiological and psychological needs of preterm infants, enhance their feeding readiness, promote recovery, and shorten the time required to achieve oral feeding^(7,8)^. However, clinical research evaluating the effectiveness of this integrated model remains limited. Therefore, this study aims to explore its potential benefits in improving feeding outcomes and overall care for preterm infants.

## METHOD

### Subjects

A total of 100 preterm infants hospitalized for treatment herein between January 2022 and January 2024 were recruited using a convenience sampling method based on the total number of eligible admissions during that period. These infants were randomly assigned into a control group (n = 50) and an intervention group (n = 50) based on different nursing intervention protocols. This study was approved by the institutional ethics committee of Changzhou Maternal and Child Health Care Hospital (no. JSCZMH20221054). To minimize assessment bias, the outcome assessors were blinded to group assignments throughout the data analysis process. In the control group, there were 27 males and 23 females, with a gestational age of 28–32 (30.15 ± 1.02) weeks, weight of preterm infants of 1300–2400 (1800 ± 250) g, and maternal age of 26–34 (30.11 ± 1.14) years old. Regarding the mode of delivery, 28 cases of spontaneous delivery and 22 cases of cesarean delivery were recorded. The intervention group consisted of 26 males and 24 females, and the gestational age, weight of preterm infants, and maternal age were 28–32 (30.14 ± 1.01) weeks, 1300–2400 (1750 ± 260) g, and 26–34 (30.13 ± 1.16) years old, respectively. The mode of delivery was recorded as spontaneous delivery in 27 cases and cesarean delivery in 23 cases. The basic data were comparable between the two groups (P > 0.05).

The inclusion criteria were: 1) preterm infants clinically diagnosed based on signs and symptoms, 2) gestational age < 37 weeks and birth weight < 2500 g, 3) parents with good physical and mental health, and 4) informed consent signed by the parents.

The under-mentioned exclusion criteria included: 1) preterm infants with congenital diseases, 2) family history of mental diseases, or 3) severe dysfunction of vital organs.

### Ethical Aspects Subsection

#### Conventional Nursing Intervention for Control Group

Warm nursing: Incubators with good humidifying and heating function were utilized to place the preterm infants, in which the temperature and humidity were adjusted according to the specific weight and gestational age of infants, and the indoor temperature and relative humidity were maintained at 24–26°C and 55–65%, respectively. The umbilical part of preterm infants was kept dry and clean. Nursing staff were required to wash hands before touching preterm infants and after changing diapers. Milk utensils were carefully sterilized and cleaned after each feeding, and the room was ventilated every day.

Feeding nursing: Breast feeding was the main choice, the daily feeding amount was determined *as per* the individual qualities of preterm infants, and the preterm infants were fed in small amounts at higher frequencies. The intake of nutrients (110 cal/kg) and proteins (accounting for 10.2% of total calories) was increased according to the demands of preterm infants. Besides, vitamins C, D, E and iron were timely supplemented to the preterm infants with anemia and hypovitaminosis. Vitamin D supplementation started from the second day after birth.

Conventional nursing: After entering the intensive care unit, the preterm infants were monitored for blood oxygen saturation and respiration, their respiratory tract was cleaned promptly, and they were gently stimulated to observe the respiration, apnea duration and skin color.

### Multi-Dimensional Sensory Touch and Family-Accompanied Whole-Process Nursing System for Intervention Group

#### Team Creation

A total of four nursing staff members with more than four years of clinical experience were selected from the Department of Neonatology to form an intervention team. The team included one charge nurse as the leader, who was responsible for drafting, revising, and finalizing the intervention protocol. Knowledge training sessions were conducted by the team leader every five days. Relevant books were used to explain the principles of family-accompanied whole-process nursing (nursing measures and precautions) and multi-dimensional sensory touch nursing. Weekly assessments were performed using a 100-point questionnaire (≥85 points required to pass) to ensure all staff achieved proficiency.

### Protocol Implementation

(1) Protocol development: On the day of admission, team members conducted a 10-minute face-to-face health education session with the families of preterm infants. Basic demographic and clinical information (name, gender, general health status, gestational age, birth weight) was collected through structured interviews and recorded in individual case files. Based on successful prior cases and standardized hospital procedures, individualized intervention protocols were developed for each infant, incorporating multi-sensory stimulation (hearing, vision, smell, taste, touch, and vestibular sense). The protocols were adjusted according to the infant’s condition, gestational age, and tolerance to external stimuli.
(2) Training and NICU entry: After protocol formulation, the team leader organized a 15-minute training session using a professionally produced educational video that explained the concepts and procedures of multi-dimensional sensory touch and family-accompanied whole-process care. Verbal explanations and live demonstrations were provided, focusing on key skills such as proper diaper changing, feeding posture, and safe handling. Following training, families were guided into the neonatal intensive care unit (NICU) under strict infection control protocols (handwashing, wearing isolation gowns, masks, and caps). To ensure consistency, family participation in the NICU was standardized: each family accompanied their infant at least four times per week, with each session lasting more than three hours (average duration 4.2 ± 0.6 hours). Families received hands-on training under professional supervision, with nursing staff correcting improper techniques and ensuring accurate implementation of each sensory stimulation step.
(3) Vision intervention: During wakeful periods, visual engagement was provided using colored rubber balls (yellow, blue, or red). A ball was selected, placed 20–40 cm above the infant’s eyes, and gently shaken to attract visual attention. Once engaged, the ball was slowly moved horizontally for 3–5 minutes. During feeding, families and nursing staff maintained smiles and gentle eye contact, conveying warmth and encouragement to promote visual comfort and enhance the infant’s acceptance of feeding.
(4) Hearing intervention: Soothing, soft sounds such as classical lullabies and nature sounds (e.g., ocean waves, birdsong) were played 10–15 minutes before feeding, with the volume kept at 50 dB to avoid overstimulation. During feeding, nursing staff spoke softly to the infants, while families were encouraged to call the infants’ names and gently encourage them (e.g., “Have a meal, my baby”), enhancing auditory familiarity and a sense of security.
(5) Touch intervention: Before or between feedings, families—after proper hand hygiene—gently massaged the infants’ cheeks, lips, and chin with moderate pressure, moving from the outer to inner cheeks and from the periphery to the center of the lips. This was followed by whole-body massage (abdomen, chest, limbs, and back) while the hands remained warm. Each session lasted 15 minutes, performed twice daily. Pressure was standardized to ensure warmth, comfort, and continuity of contact.
(6) Smell intervention: Specialist nurses provided guidance for olfactory stimulation. Families rewarmed pre-prepared breast milk to about 40 °C, evenly dripped it onto sterile gauze, and fully moistened the gauze. The gauze was then held about 4 cm from the infant’s nostrils for two minutes per session, three times daily (morning, noon, and evening). This aimed to stimulate olfactory memory and feeding reflexes through familiar maternal scents.
(7) Vestibular intervention: Nursing staff slowly held the infant against the front of the body, supporting the head and back so the infant remained aligned. The infant was gently swayed horizontally once every three seconds, twice daily. Movements were slow, even, and closely monitored to ensure safety and comfort.

### Evaluation of Indicators for Feeding Process

The indicators for the feeding process of preterm infants in the two groups, including transition time to oral feeding, extubation time of gastric tube and length of hospital stay, were recorded in detail and compared by the nursing staff.

### Assessment Using Neonatal Behavioral Neurological Assessment Scale (NBNA)

On the day of admission and at 1 d before discharge, the nursing staff assessed the growth and development of the preterm infants in both groups. The scale contained a total of 20 items under 5 entries, namely, passive muscular tension (4 indicators), primitive reflexes (3 indicators), general evaluation (3 indicators), active muscular tension (4 indicators), and behavioral ability (6 indicators). Each item was scored 3 points, with a total score of 60 points, and a higher score signified better behavioral and neurological development. The Cronbach’s coefficient of the scale was 0.871, the split-half reliability was 0.855, and the validity was good.

### Assessment Using Apgar Scale

The physical signs of the two groups of preterm infants were assessed and compared by the nursing staff on the day of admission and one day before discharge. The assessment covered five dimensions: pulse, frowning response, reaction to stimuli, skin color, and respiration. Each dimension was scored out of 5 points, for a total of 25 points. A higher score indicated a better health status of the preterm infants. The reliability of the scale was demonstrated by Cronbach’s alpha (0.876), split-half reliability (0.876), and test–retest reliability (0.844), indicating good reliability and validity.

### Statistical Analysis

Data were analyzed using SPSS 26.0 software. Categorical data were presented as [n (%)] and compared using the χ^2^ test. Normally distributed measurement data (feeding process, NBNA score, and Apgar score) were expressed as mean ± standard deviation ( $\bar{\text{X}}$ ± s). Intergroup comparisons were con­ducted using a two-group parallel design, while intragroup comparisons before and after treatment were performed using a paired t-test. A p-value of < 0.05 was considered statistically significant.

## RESULTS

### Indicators for Feeding Process

The intervention group showed significantly shorter transition time to oral feeding, gastric tube extubation time, and length of hospital stay compared to the control group (P < 0.05) (Table 1).

**Table 1:** Indicators for feeding process ( $\bar{\text{X}}$ ± s, d) – Changzhou, Jiangsu Province, China, 2022.

Group	Transition time to oral feeding	Extubation time of gastric tube	Length of hospital stay
Observation (n = 50)	4.93 ± 0.45	7.85 ± 0.77	22.44 ± 1.75
Control (n = 50)	5.27 ± 0.50	8.21 ± 0.95	23.44 ± 2.83
*t*	3.574	2.082	2.125
P	0.001	0.040	0.036

NBNA scores.

On the day of admission, no significant difference was detected in the NBNA score between the two groups (P > 0.05). The NBNA score was increased in the intervention group compared with that in the control group at follow-up, showing a significant difference (P < 0.05) (Table 2).

**Table 2:** NBNA scores ( $\bar{\text{X}}$ ± s, point) – Changzhou, Jiangsu Province, China, 2022.

Group	Passive muscular tension		Primitive reflex		General evaluation	
	On the day of admission	1 d before discharge	On the day of admission	1 d before discharge	On the day of admission	1 d before discharge
Observation (n = 50)	6.55 ± 0.38	9.12 ± 0.66*	4.45 ± 0.22	6.80 ± 0.31*	4.73 ± 0.26	6.58 ± 0.32*
Control (n = 50)	6.51 ± 0.40	8.73 ± 0.51*	4.51 ± 0.21	6.59 ± 0.29*	4.81 ± 0.24	6.39 ± 0.29*
*t*	0.513	3.306	1.395	3.498	1.599	3.111
P	0.609	0.001	0.166	0.001	0.113	0.002
**Group**	**Active muscular tension**		**Behavioral ability**		**Total score**	
	**On the day of admission**	**1 d before discharge**	**On the day of admission**	**1 d before discharge**	**On the day of admission**	**1 d before discharge**
Observation (n = 50)	6.39 ± 0.41	8.99 ± 0.45*	10.74 ± 0.66	14.63 ± 1.02*	35.11 ± 1.32	43.52 ± 1.60*
Control (n = 50)	6.41 ± 0.40	8.71 ± 0.39*	10.81 ± 0.61	13.96 ± 0.98*	35.42 ± 1.30	42.51 ± 1.54*
*t*	0.224	8.572	0.499	6.036	1.183	3.216
P	0.824	< 0.01	0.619	< 0.01	0.240	0.002

*P < 0.05 *vs.* the same group on the day of admission. NBNA: Neonatal Behavioral Neurological Assessment Scale.

### Apgar Scores

The difference in Apgar score was not significant between the two groups on the day of admission (P > 0.05). At 1 d before discharge, an increase in the Apgar score was detected from the intervention group by contrast to that in the control group, with a difference of significance (P < 0.05) (Table 3).

**Table 3:** Apgar score ( $\bar{\text{X}}$ ± s, point) – Changzhou, Jiangsu Province, China, 2022.

Group	Pulse		Frowning action		Stimulus response	
	On the day of admission	1 d before discharge	On the day of admission	1 d before discharge	On the day of admission	1 d before discharge
Observation (n = 50)	3.24 ± 0.43	3.87 ± 0.68*	3.64 ± 0.41	4.27 ± 0.69*	4.11 ± 0.63	4.99 ± 0.82*
Control (n = 50)	3.26 ± 0.41	3.57 ± 0.55*	3.59 ± 0.41	3.88 ± 0.57*	4.13 ± 0.60	4.44 ± 0.78*
*t*	0.238	2.426	0.610	3.081	0.163	3.436
P	0.812	0.017	0.543	0.003	0.871	0.001
**Group**	**Skin color**		**Respiration**		**Total score**	
	**On the day of admission**	**1 d before discharge**	**On the day of admission**	**1 d before discharge**	**On the day of admission**	**1 d before discharge**
Observation (n = 50)	3.17 ± 0.38	3.79 ± 0.64*	3.17 ± 0.38	3.79 ± 0.64*	15.32 ± 1.21	17.57 ± 1.55*
Control (n = 50)	3.15 ± 0.35	3.44 ± 0.52*	3.15 ± 0.35	3.44 ± 0.52*	15.41 ± 1.19	16.59 ± 1.49*
*t*	0.274	3.001	0.274	3.001	0.375	3.223
P	0.785	0.003	0.785	0.003	0.708	0.002

*P < 0.05 *vs.* the same group on the day of admission. Apgar: Apgar Scale.

## DISCUSSION

As a group of special newborns, preterm infants face many serious challenges in feeding due to the immature development of various organs and systems, particularly the underdeveloped digestive system^(9)^. According to data from the World Health Organization, the global birth rate of preterm infants is increasing, and feeding difficulties—along with related complications—are major contributing factors to growth retardation and increased health problems in this population^(10)^. Despite advancements in perinatology in China and improvements in the survival rates of preterm infants, feeding-related issues remain significant clinical concerns that affect prognosis.

Traditional feeding strategies have primarily focused on meeting basic nutritional requirements, often overlooking the heightened need for sensory stimulation and emotional support, both of which play crucial roles in neurodevelopment and physiological regulation. In recent years, the concept of multi-dimensional sensory touch nursing has attracted growing attention. This approach involves targeted stimulation of the senses, such as vision, hearing, touch, smell, and taste, to promote neural development, enhance physiological function, and ultimately improve feeding outcomes^(11)^. Meanwhile, the family-accompanied whole-process nursing model emphasizes continuous, family-centered care that addresses both physical and emotional needs throughout hospitalization^(12)^. Integrating these two approaches creates a comprehensive and humanized care system tailored to the unique developmental characteristics of preterm infants.

In this study, the integrated nursing approach significantly shortened the transition time to oral feeding, reduced gastric tube extubation time, and decreased the length of hospital stay in the intervention group compared with the control group (P < 0.05). Visually, the combination of multi-dimensional sensory touch and family-accompanied whole-process nursing helped reduce stress responses in preterm infants and stimulated their interest in feeding through the use of soft lighting and brightly colored visual aids. In addition, the smiles and eye contact of nursing staff throughout the process conveyed warmth and care, enhancing feeding acceptance, promoting coordination of sucking and swallowing, and improving feeding efficiency^(13)^.

In terms of auditory stimulation, soft music helped regulate the nervous system, relieve anxiety, promote digestive juice secretion, and support digestion and absorption. Gentle speech from nursing staff also contributed to a positive feeding experience, improving cooperation and facilitating smoother feeding processes. Regarding olfactory stimulation, the scent of breast milk activated the rooting reflex, increased the desire to feed, and enhanced feeding success. Furthermore, a personalized feeding protocol ensured that nutritional needs were met, while a variety of taste experiences supported the development of the sense of taste and improved food acceptance^(14,15)^.

The study found that NBNA and Apgar scores were significantly higher in the intervention group than in the control group (P < 0.05), indicating improved neurological and physiological development in the preterm infants. The enhancements in feeding, driven by multi-dimensional sensory touch, directly increased nutritional intake, which provided a solid foundation for overall growth and development. Moreover, a comfortable sensory environment and appropriate feeding methods facilitated gastrointestinal motility and digestive juice secretion, improving the efficiency of nutrient digestion and absorption. These improvements were favorable for weight gain and the development of various organs and tissues^(16,17)^.

Additionally, diverse sensory stimulation supported nervous system development. Stimulation of the visual, auditory, and tactile senses enriched synaptic connections in the brain, promoting neurological development. A well-developed nervous system helped preterm infants better control body movements, coordinate swallowing and breathing, and further enhance growth and development^(18)^.

The study also demonstrated that a comfortable sensory environment, combined with the presence of family and attentive nursing care, reduced stress responses and stabilized respiration in preterm infants. For instance, soft music and skin-to-skin contact helped alleviate anxiety and prevent irregular breathing caused by emotional fluctuations. Adequate nutritional intake and proper circulatory function ensured good blood supply to the skin, resulting in a healthy, rosy complexion. In this way, a supportive sensory environment and effective nursing interventions improved overall health status and physical signs in preterm infants^(19,20)^.

Although this study provides solid and reliable findings, it has several limitations. The sample size was relatively small, and all participants were recruited from a single study site, which may introduce selection bias. Future research should aim to expand the sample size and include a broader range of observation indicators to validate and enrich the current results. Additionally, the use of a convenience sample—based on all eligible admissions during the study period—may limit the generalizability of the findings. Since participants were not randomly selected from a larger population, the possibility of selection bias cannot be entirely ruled out. Therefore, caution should be exercised when applying these findings to other populations or clinical settings.

## CONCLUSION

In conclusion, multi-dimensional sensory touch plus family-accompanied whole-process nursing system in the feeding intervention for preterm infants demonstrates significant nursing efficacy while improving the growth, development, and feeding process.

## Data Availability

The entire dataset supporting the results of this study was published in the article itself.

## References

[1] Dunne EA, O’Donnell CPF, Nakstad B, McCarthy LK (2024). Thermoregulation for very preterm infants in the delivery room: a narrative review.. Pediatr Res.

[2] Barrett-Reis B, Shakeel F, Dennis L, Baggs G, Masor ML (2023). Acidified feedings in preterm infants: a historical and physiological perspective.. Am J Perinatol.

[3] Kyler H (2024). The multisensory and multidimensional nature of object representation.. J Neurophysiol.

[4] Sgueglia GA, Hassan A, Harb S, Ford TJ, Koliastasis L, Milkas A (2022). International hand function study following distal radial access: the RATATOUILLE study.. JACC Cardiovasc Interv.

[5] Hu F, Zhou Q, Liu R, Zhu Y, Liang Y, Fang D (2025). Top-down architecture of magnetized micro-cilia and conductive micro-domes as fully bionic electronic skin for de-coupled multidimensional tactile perception.. Mater Horiz.

[6] Peery AF, Crockett SD, Murphy CC, Jensen ET, Kim HP, Egberg MD (2022). Burden and cost of gastrointestinal, liver, and pancreatic diseases in the United States: update 2021.. Gastroenterology.

[7] Bschor T, Nagel L, Unger J, Schwarzer G, Baethge C (2024). Differential outcomes of placebo treatment across 9 psychiatric disorders: a systematic review and meta-analysis.. JAMA Psychiatry.

[8] Liu M, Dai Z, Zhao Y, Ling H, Sun L, Lee C (2024). Tactile sensing and rendering patch with dynamic and static sensing and haptic feedback for immersive communication.. ACS Appl Mater Interfaces.

[9] Glenzel L, do Nascimento Oliveira P, Marchi BS, Ceccon RF, Moran CA (2023). Validity and reliability of pain and behavioral scales for preterm infants: a systematic review.. Pain Manag Nurs.

[10] Linnér A, Lode Kolz K, Klemming S, Bergman N, Lilliesköld S, Markhus Pike H (2022). Immediate skin-to-skin contact may have beneficial effects on the cardiorespiratory stabilisation in very preterm infants.. Acta Paediatr.

[11] Thomas JP, Völter C, Wirth R, Guthoff R, Grunwald M, Hummel T (2021). How the brain perceives the world in old age with all senses.. Z Gerontol Geriatr.

[12] Joo KA, Fialkowski MK, Esquivel M, Haumea SL (2022). Hawai’i registered dietitian nutritionist 2019-2020 workforce assessment.. Hawaii J Health Soc Welf.

[13] Davis MJ, Luu BC, Raj S, Abu-Ghname A, Buchanan EP (2021). Multidisciplinary care in surgery: are team-based interventions cost-effective?. Surgeon.

[14] Kawazoe A, Reardon G, Woo E, Luca MD, Visell Y (2021). Tactile echoes: multisensory augmented reality for the hand.. IEEE Trans Haptics.

[15] Raj S, Williams EM, Davis MJ, Abu-Ghname A, Luu BC, Buchanan EP (2021). Cost-effectiveness of multidisciplinary care in plastic surgery: a systematic review.. Ann Plast Surg.

[16] Khamis H, Afzal HMN, Sanchez J, Vickery R, Wiertlewski M, Redmond SJ (2021). Friction sensing mechanisms for perception and motor control: passive touch without sliding may not provide perceivable frictional information.. J Neurophysiol.

[17] Feldens CA, Heck ABDS, Rodrigues PH, Coelho EMRB, Vítolo MR, Kramer PF (2024). Ankyloglossia and breastfeeding duration: a multicenter birth cohort study.. Breastfeed Med.

[18] Kim S, Lim A, Jang H, Jeon M (2023). Life-sustaining treatment decision in palliative care based on electronic health records analysis.. J Clin Nurs.

[19] Zhang R, Schwehr TJ, Abbott JJ (2020). Dimensional reduction for 6d vibrotactile display.. IEEE Trans Haptics.

[20] Yasin ZM, Anderson PD, Lingman M, Kwatra J, Ashfaq A, Slutzman JE (2021). Receiving care according to national heart failure guidelines is associated with lower total costs: an observational study in Region Halland, Sweden.. Eur Heart J Qual Care Clin Outcomes.

