# Supplementary material for: Effect of multi-dimensional sensory touch and family-accompanied whole-process nursing on feeding intervention for preterm infants
Source: Rev Esc Enferm USP. 2025 Nov 21;59:e20250128. doi: 10.1590/1980-220X-REEUSP-2025-0128en (PMC12668600; doi:10.1590/1980-220X-REEUSP-2025-0128en)
Supplement: Translation

# Translation: Efecto del tacto sensorial multidimensional y la atención de enfermería integral acompañada por la familia en la intervención alimentaria para bebés prematuros

**DOI:** 10.1590/1980-220X-REEUSP-2025-0128es

**Authors:** Ting Ye, Lijuan Gao

**Affiliations:** 1 Changzhou Maternal and Child Health Care Hospital, Changzhou, Jiangsu Province, China.

**Keywords:** Familia, Dieta, Enfermería, Recien Nacido Prematuro, Tacto

## Abstract

**Objetivo:**

Nuestro objetivo fue evaluar el efecto del toque sensorial multidimensional y del cuidado de enfermería como proceso integral acompañado por la familia en la intervención de alimentación de recién nacidos prematuros.

**Métodos:**

Se diseñó un estudio experimental retrospectivo para reclutar a 100 recién nacidos prematuros ingresados y tratados entre enero de 2022 y enero de 2024 como sujetos, con un grupo de intervención (n = 50, sometidos al toque sensorial multidimensional en el marco del cuidado de enfermería como proceso integral acompañado por la familia) y un grupo control (n = 50, que recibió enfermería convencional), establecidos de acuerdo con diferentes protocolos de enfermería.

**Resultados:**

El grupo de intervención presentó tiempos significativamente menores de transición a la alimentación oral, de retirada de la sonda gástrica y de estancia hospitalaria que el grupo control (P < 0,05). La puntuación de la *Neonatal Behavioral Neurological Assessment Scale* (NBNA) aumentó significativamente en el grupo de intervención en comparación con la del grupo control (P < 0,05). Un día antes del alta, se detectó un aumento significativo en la puntuación de Apgar en el grupo de intervención respecto al grupo control (P < 0,05).

**Conclusión:**

El toque sensorial multidimensional y la lactancia completa acompañada por la familia pueden mejorar significativamente los signos físicos generales de los recién nacidos prematuros y facilitar gradualmente su desarrollo.

## INTRODUCCIÓN

Los bebés prematuros, nacidos antes de alcanzar la edad equivalente al término, suelen presentar un desarrollo inmaduro de diversos órganos y sistemas. Esta inmadurez da lugar a diferencias notables tanto en el aspecto como en las funciones fisiológicas en comparación con los bebés nacidos a término. En particular, el subdesarrollo de los sistemas respiratorio, inmunológico y termorregulador los hace especialmente vulnerables a complicaciones como la hipoxia, la inestabilidad de la temperatura corporal y el aumento del riesgo de infección, lo que plantea importantes retos para la atención clínica y la enfermería^(1–3)^. Además, los recursos médicos limitados y la elevada carga de trabajo en los entornos pediátricos pueden comprometer la puntualidad y la calidad de la atención de enfermería convencional, lo que afecta negativamente al pronóstico y la supervivencia de estos bebés vulnerables.

Para hacer frente a estos retos, ha surgido el tacto sensorial multidimensional como enfoque terapéutico que involucra múltiples sentidos—incluidos la vista, el oído, el tacto, el olfato y el gusto—para evocar experiencias perceptivas integradas y respuestas emocionales tanto a nivel fisiológico como psicológico, lo que favorece el desarrollo del bebé^(4,5)^. Al mismo tiempo, se ha demostrado que la enfermería integral acompañada por la familia, un modelo de atención centrado en el paciente que hace hincapié en la participación continua de la familia y la atención integral, mejora la comodidad y la estabilidad en la atención neonatal^(6)^.

Recientemente, los médicos han comenzado a integrar estas dos intervenciones en las estrategias de alimentación, ofreciendo tanto estimulación sensorial como apoyo familiar constante durante toda la estancia hospitalaria. Este enfoque combinado tiene como objetivo abordar las necesidades fisiológicas y psicológicas de los bebés prematuros, mejorar su disposición para la alimentación, promover la recuperación y acortar el tiempo necesario para lograr la alimentación oral^(7,8)^. Sin embargo, las investigaciones clínicas que evalúan la eficacia de este modelo integrado siguen siendo limitadas. Por lo tanto, este estudio tiene como objetivo explorar sus posibles beneficios para mejorar los resultados de la alimentación y la atención general de los bebés prematuros.

## MÉTODO

### Sujetos

Se reclutó a un total de 100 bebés prematuros hospitalizados para recibir tratamiento entre enero de 2022 y enero de 2024, utilizando un método de muestreo por conveniencia basado en el número total de ingresos elegibles durante ese periodo. Estos bebés fueron asignados aleatoriamente a un grupo de control (n = 50) y a un grupo de intervención (n = 50) en función de diferentes protocolos de intervención de enfermería. Este estudio fue aprobado por el comité de ética institucional del Hospital de Atención de Salud Materno-Infantil de Changzhou (n.º JSCZMH20221054). Para minimizar el sesgo de evaluación, los evaluadores de resultados desconocían la asignación de los grupos durante todo el proceso de análisis de datos. En el grupo de control, había 27 varones y 23 mujeres, con una edad gestacional de 28–32 (30,15 ± 1,02) semanas, un peso de los bebés prematuros de 1300–2400 (1800 ± 250) g y una edad materna de 26–34 (30,11 ± 1,14) años. En cuanto al tipo de parto, se registraron 28 casos de parto espontáneo y 22 casos de cesárea. El grupo de intervención estaba formado por 26 varones y 24 mujeres, y la edad gestacional, el peso de los bebés prematuros y la edad materna eran de 28–32 (30,14 ± 1,01) semanas, 1300–2400 (1750 ± 260) g y 26–34 (30,13 ± 1,16) años, respectivamente. El modo de parto se registró como parto espontáneo en 27 casos y parto por cesárea en 23 casos. Los datos básicos eran comparables entre los dos grupos (P > 0,05).

Los criterios de inclusión fueron: 1) bebés prematuros diagnosticados clínicamente en función de los signos y síntomas, 2) edad gestacional < 37 semanas y peso al nacer < 2500 g, 3) padres con buena salud física y mental, y 4) consentimiento informado firmado por los padres.

Los criterios de exclusión mencionados a continuación incluyeron: 1) bebés prematuros con enfermedades congénitas, 2) antecedentes familiares de enfermedades mentales, o 3) disfunción grave de órganos vitales.

### Subsección Aspectos Éticos

#### Intervención de Enfermería Convencional para el Grupo de Control

Cuidados de calor: se utilizaron incubadoras con buena función de humidificación y calefacción para colocar a los bebés prematuros, en las que se ajustó la temperatura y la humedad según el peso específico y la edad gestacional de los bebés, y se mantuvieron la temperatura interior y la humedad relativa entre 24 y 26 °C y entre 55 y 65%, respectivamente. Se mantuvo seca y limpia la parte umbilical de los bebés prematuros. El personal de enfermería debía lavarse las manos antes de tocar a los bebés prematuros y después de cambiarles los pañales. Los utensilios para la leche se esterilizaban y limpiaban cuidadosamente después de cada toma, y la habitación se ventilaba todos los días.

Atención de alimentación: La lactancia materna era la opción principal, la cantidad diaria de alimentación se determinaba*según*las cualidades individuales de los bebés prematuros, y estos se alimentaban en pequeñas cantidades y con mayor frecuencia. La ingesta de nutrientes (110 cal/kg) y proteínas (que representaban el 10,2% del total de calorías) se aumentó de acuerdo con las necesidades de los bebés prematuros. Además, se administraron suplementos de vitaminas C, D y E y hierro a los bebés prematuros con anemia e hipovitaminosis. La administración de suplementos de vitamina D comenzó a partir del segundo día después del nacimiento.

Cuidados convencionales: tras ingresar en la unidad de cuidados intensivos, se monitoreó la saturación de oxígeno en sangre y la respiración de los bebés prematuros, se les limpió rápidamente las vías respiratorias y se les estimuló suavemente para observar la respiración, la duración de la apnea y el color de la piel.

### Sistema de Cuidados Multidimensionales Sensoriales y de Acompañamiento Familiar Durante Todo el Proceso para el Grupo de Intervención

#### Creación del Equipo

Se seleccionó a un total de cuatro miembros del personal de enfermería con más de cuatro años de experiencia clínica del Departamento de Neonatología para formar un equipo de intervención. El equipo incluía a una enfermera jefe como líder, que se encargaba de redactar, revisar y finalizar el protocolo de intervención. El líder del equipo impartía sesiones de formación cada cinco días. Se utilizaron libros relevantes para explicar los principios de la enfermería integral acompañada por la familia (medidas y precauciones de enfermería) y la enfermería sensorial multidimensional. Se realizaron evaluaciones semanales mediante un cuestionario de 100 puntos (se requería una puntuación mínima de 85 puntos para aprobar) con el fin de garantizar que todo el personal alcanzara el nivel de competencia necesario.

### Aplicación Del Protocolo

(1) Desarrollo del protocolo: El día del ingreso, los miembros del equipo llevaron a cabo una sesión presencial de educación sanitaria de 10 minutos con las familias de los bebés prematuros. Para recolectar información demográfica y clínica básica (nombre, sexo, estado de salud general, edad gestacional, peso al nacer), se realizaron entrevistas estructuradas y se registró en expedientes individuales. Basándose en casos anteriores exitosos y en procedimientos hospitalarios estandarizados, se desarrollaron protocolos de intervención individualizados para cada bebé, incorporando estimulación multisensorial (audición, visión, olfato, gusto, tacto y sentido vestibular). Los protocolos se ajustaron según el estado del bebé, la edad gestacional y la tolerancia a los estímulos externos.
(2) Capacitación y entrada en la UCIN: Tras la formulación del protocolo, el jefe del equipo organizó una sesión de capacitación de 15 minutos utilizando un video educativo producido profesionalmente que explicaba los conceptos y procedimientos del tacto sensorial multidimensional y la atención durante todo el proceso acompañada por la familia. Se proporcionaron explicaciones verbales y demostraciones en vivo, centrándose en habilidades clave como el cambio adecuado de pañales, la postura para alimentar y la manipulación segura. Tras la capacitación, se guio a las familias a la unidad de cuidados intensivos neonatales (UCIN) bajo estrictos protocolos de control de infecciones (lavado de manos, uso de batas de aislamiento, cubrebocas y gorros). Para garantizar la coherencia, se estandarizó la participación de las familias en la UCIN: cada familia acompañó a su bebé al menos cuatro veces por semana, con una duración de más de tres horas por sesión (duración media de 4,2 ± 0,6 horas). Las familias recibieron formación práctica bajo supervisión profesional, y el personal de enfermería corrigió las técnicas inadecuadas y garantizó la aplicación correcta de cada paso de la estimulación sensorial.
(3) Intervención visual: durante los periodos de vigilia, se proporcionó estimulación visual utilizando pelotas de goma de colores (amarillas, azules o rojas). Se seleccionaba una pelota, se colocaba a 20–40 cm por encima de los ojos del bebé y se agitaba suavemente para atraer su atención visual. Una vez captada su atención, la pelota se movía lentamente en horizontal durante 3–5 minutos. Durante la alimentación, las familias y el personal de enfermería mantenían sonrisas y un contacto visual suave, transmitiendo calidez y ánimo para promover la comodidad visual y mejorar la aceptación de la alimentación por parte del bebé.
(4) Intervención auditiva: Se reprodujeron sonidos suaves y relajantes, como canciones de cuna clásicas y sonidos de la naturaleza (por ejemplo, olas del mar, canto de pájaros), entre 10 y 15 minutos antes de la alimentación, manteniendo el volumen a 50 dB para evitar la sobreestimulación. Durante la alimentación, el personal de enfermería hablaba suavemente a los bebés, mientras que se animaba a las familias a llamar a los bebés por su nombre y animarlos con delicadeza (por ejemplo, «Come, mi bebé»), lo que mejoraba la familiaridad auditiva y la sensación de seguridad.
(5) Intervención táctil: antes o entre las tomas, las familias, tras una higiene adecuada de las manos, masajeaban suavemente las mejillas, los labios y el mentón de los bebés con una presión moderada, moviéndose desde el exterior hacia el interior de las mejillas y desde la periferia hacia el centro de los labios. A continuación, se realizaba un masaje de todo el cuerpo (abdomen, pecho, extremidades y espalda) mientras las manos permanecían calientes. Cada sesión duraba 15 minutos y se realizaba dos veces al día. La presión se estandarizó para garantizar el calor, la comodidad y la continuidad del contacto.
(6) Intervención olfativa: enfermeras especializadas proporcionaron orientación para la estimulación olfativa. Las familias recalentaron la leche materna preparada previamente a unos 40 °C, la vertieron uniformemente sobre una gasa estéril y la humedecieron por completo. A continuación, la gasa se mantuvo a unos 4 cm de las fosas nasales del bebé durante dos minutos por sesión, tres veces al día (por la mañana, al mediodía y por la noche). El objetivo era estimular la memoria olfativa y los reflejos de alimentación a través de los aromas familiares de la madre.
(7) Intervención vestibular: El personal de enfermería sostenía lentamente al bebé contra la parte delantera del cuerpo, apoyando la cabeza y la espalda para que el bebé permaneciera alineado. Se balanceaba suavemente al bebé en horizontal una vez cada tres segundos, dos veces al día. Los movimientos eran lentos, uniformes y se monitoreaban de cerca para garantizar la seguridad y la comodidad.

### Evaluación de los Indicadores del Proceso de Alimentación

El personal de enfermería registró detalladamente y comparó los indicadores del proceso de alimentación de los bebés prematuros de los dos grupos, incluyendo el tiempo de transición a la alimentación oral, el tiempo de extubación de la sonda gástrica y la duración de la estadía hospitalaria.

### Evaluación Mediante la Escala de Evaluación Neurológica del Comportamiento Neonatal Neonatal Behavioral Neurological Assessment Scale (NBNA)

El día del ingreso y un día antes del alta, el personal de enfermería evaluó el crecimiento y el desarrollo de los bebés prematuros de ambos grupos. La escala contenía un total de 20 ítems en 5 entradas, a saber, tensión muscular pasiva (4 indicadores), reflejos primitivos (3 indicadores), evaluación general (3 indicadores), tensión muscular activa (4 indicadores) y capacidad conductual (6 indicadores). Cada ítem se puntuó con 3 puntos, con una puntuación total de 60 puntos, y una puntuación más alta significaba un mejor desarrollo conductual y neurológico. El coeficiente de Cronbach de la escala fue de 0,871, la fiabilidad de la mitad dividida fue de 0,855 y la validez fue buena.

### Evaluación Mediante la Escala de Apgar

El personal de enfermería evaluó y comparó los signos físicos de los dos grupos de bebés prematuros el día del ingreso y un día antes del alta. La evaluación abarcó cinco dimensiones: pulso, respuesta al fruncimiento del ceño, reacción a los estímulos, color de la piel y respiración. Cada dimensión se puntuó con un máximo de 5 puntos, para un total de 25 puntos. Una puntuación más alta indicaba un mejor estado de salud de los bebés prematuros. La fiabilidad de la escala quedó demostrada por el alfa de Cronbach (0,876), la fiabilidad de la mitad dividida (0,876) y la fiabilidad test-retest (0,844), lo que indica una buena fiabilidad y validez.

### Análisis Estadístico

Los datos se analizaron utilizando el software SPSS 26.0. Los datos categóricos se presentaron como [n (%)] y se compararon utilizando la prueba χ^2^. Los datos de medición distribuidos normalmente (proceso de alimentación, puntuación NBNA y puntuación Apgar) se expresaron como media ± desviación estándar ( $\bar{\text{X}}$ ± s). Las comparaciones entre grupos se realizaron utilizando un diseño paralelo de dos grupos, mientras que las comparaciones intragrupo antes y después del tratamiento se realizaron utilizando una prueba t pareada. Se consideró estadísticamente significativo un valor p < 0,05.

## RESULTADOS

### Indicadores del Proceso de Alimentación

El grupo de intervención mostró un tiempo de transición a la alimentación oral, un tiempo de extubación de la sonda gástrica y una duración de la estadía significativamente más cortos en comparación con el grupo de control (P < 0,05) (Tabla 1).

**Tabla 1:** Indicadores del proceso de alimentación (scores ( $\bar{\text{X}}$ ± s, d) – Changzhou, provincia de Jiangsu, China, 2022.

Grupos	Tiempo de transición a alimentación oral	Tiempo de extubación de tubo gástrico	Duración de estadía hospitalaria
Observación (n = 50)	4.93 ± 0.45	7.85 ± 0.77	22.44 ± 1.75
Control (n = 50)	5.27 ± 0.50	8.21 ± 0.95	23.44 ± 2.83
*t*	3.574	2.082	2.125
P	0.001	0.040	0.036

Puntuación NBNA.

El día del ingreso, no se detectaron diferencias significativas en la puntuación NBNA entre los dos grupos (P > 0,05). La puntuación NBNA aumentó en el grupo de intervención en comparación con la del grupo de control en el seguimiento, mostrando una diferencia significativa (P < 0,05) (Tabla 2).

**Tabla 2:** Puntuaciones NBNA ( $\bar{\text{X}}$ ± s, puntos) – Changzhou, provincia de Jiangsu, China, 2022.

Grupo	Tensión muscular pasiva		Reflejo primitivo		Evaluación general	
	En día de ingreso	1 día antes del alta	En día de ingreso	1 día antes del alta	En día de ingreso	1 día antes del alta
Observación (n = 50)	6.55 ± 0.38	9.12 ± 0.66*	4.45 ± 0.22	6.80 ± 0.31*	4.73 ± 0.26	6.58 ± 0.32*
Control (n = 50)	6.51 ± 0.40	8.73 ± 0.51*	4.51 ± 0.21	6.59 ± 0.29*	4.81 ± 0.24	6.39 ± 0.29*
*t*	0.513	3.306	1.395	3.498	1.599	3.111
P	0.609	0.001	0.166	0.001	0.113	0.002
**Grupo**	**Tensión muscular activa**		**Capacidad conductual**		**Puntuación total**	
	**En día de ingreso**	**1 día antes del alta**	**En día de ingreso**	**1 día antes del alta**	**En día de ingreso**	**1 día antes del alta**
Observación (n = 50)	6.39 ± 0.41	8.99 ± 0.45*	10.74 ± 0.66	14.63 ± 1.02*	35.11 ± 1.32	43.52 ± 1.60*
Control (n = 50)	6.41 ± 0.40	8.71 ± 0.39*	10.81 ± 0.61	13.96 ± 0.98*	35.42 ± 1.30	42.51 ± 1.54*
*t*	0.224	8.572	0.499	6.036	1.183	3.216
P	0.824	< 0.01	0.619	< 0.01	0.240	0.002

*P < 0,05 comparado con el mismo grupo el día del ingreso. NBNA: Neonatal Behavioral Neurological Assessment Scale. (Escala de Evaluación Neurológica Conductual Neonatal).

### Puntuaciones de Apgar

La diferencia en la puntuación de Apgar no fue significativa entre los dos grupos el día del ingreso (P > 0,05). Un día antes del alta, se detectó un aumento en la puntuación de Apgar en el grupo de intervención, en contraste con el grupo de control, con una diferencia significativa (P < 0,05) (Tabla 3).

**Tabla 3:** Puntuaciones de Apgar ( $\bar{\text{X}}$ ± s, puntos) – Changzhou, provincia de Jiangsu, China, 2022.

Grupo	Pulso		Fruncir el ceño		Respuesta al estímulo	
	En día de ingreso	1 día antes del alta	En día de ingreso	1 día antes del alta	En día de ingreso	1 día antes del alta
Observación (n = 50)	3.24 ± 0.43	3.87 ± 0.68*	3.64 ± 0.41	4.27 ± 0.69*	4.11 ± 0.63	4.99 ± 0.82*
Control (n = 50)	3.26 ± 0.41	3.57 ± 0.55*	3.59 ± 0.41	3.88 ± 0.57*	4.13 ± 0.60	4.44 ± 0.78*
*t*	0.238	2.426	0.610	3.081	0.163	3.436
P	0.812	0.017	0.543	0.003	0.871	0.001
**Grupo**	**Color de piel**		**Respiración**		**Puntuación total**	
	**En día de ingreso**	**1 día antes del alta**	**En día de ingreso**	**1 día antes del alta**	**En día de ingreso**	**1 día antes del alta**
Observación (n = 50)	3.17 ± 0.38	3.79 ± 0.64*	3.17 ± 0.38	3.79 ± 0.64*	15.32 ± 1.21	17.57 ± 1.55*
Control (n = 50)	3.15 ± 0.35	3.44 ± 0.52*	3.15 ± 0.35	3.44 ± 0.52*	15.41 ± 1.19	16.59 ± 1.49*
*t*	0.274	3.001	0.274	3.001	0.375	3.223
P	0.785	0.003	0.785	0.003	0.708	0.002

*P < 0,05 comparado con el mismo grupo el día del ingreso.

Apgar: Escala Apgar.

## DISCUSIÓN

Como grupo de recién nacidos especiales, los bebés prematuros se enfrentan a muchos retos graves en la alimentación debido al desarrollo inmaduro de varios órganos y sistemas, en particular el sistema digestivo subdesarrollado^(9)^. Según datos de la Organización Mundial de la Salud, la tasa mundial de natalidad de bebés prematuros está aumentando, y las dificultades de alimentación, junto con las complicaciones relacionadas, son factores importantes que contribuyen al retraso del crecimiento y al aumento de los problemas de salud en esta población^(10)^. A pesar de los avances en perinatología en China y de la mejora en las tasas de supervivencia de los bebés prematuros, los problemas relacionados con la alimentación siguen siendo una preocupación clínica importante que afecta al pronóstico.

Las estrategias de alimentación tradicionales se han centrado principalmente en satisfacer las necesidades nutricionales básicas, pasando por alto a menudo la mayor necesidad de estimulación sensorial y apoyo emocional, que desempeñan un papel crucial en el desarrollo neurológico y la regulación fisiológica. En los últimos años, el concepto de cuidados sensoriales multidimensionales ha suscitado un interés creciente. Este enfoque consiste en la estimulación específica de los sentidos, como la vista, el oído, el tacto, el olfato y el gusto, para promover el desarrollo neural, mejorar la función fisiológica y, en última instancia, mejorar los resultados de la alimentación^(11)^. Por su parte, el modelo de cuidados integrales con acompañamiento familiar hace hincapié en la atención continua y centrada en la familia, que aborda tanto las necesidades físicas como emocionales durante la hospitalización^(12)^. La integración de estos dos enfoques crea un sistema de cuidados integral y humanizado, adaptado a las características de desarrollo únicas de los bebés prematuros.

En este estudio, el enfoque de enfermería integrado acortó significativamente el tiempo de transición a la alimentación oral, redujo el tiempo de extubación de la sonda gástrica y disminuyó la duración de la estadía en el grupo de intervención en comparación con el grupo de control (P < 0,05). Visualmente, la combinación de la estimulación sensorial multidimensional y la enfermería integral acompañada por la familia ayudó a reducir las respuestas de estrés en los bebés prematuros y estimuló su interés por la alimentación mediante el uso de iluminación suave y ayudas visuales de colores vivos. Además, las sonrisas y el contacto visual del personal de enfermería durante todo el proceso transmitieron calidez y cuidado, lo que mejoró la aceptación de la alimentación, promovió la coordinación de la succión y la deglución y mejoró la eficiencia de la alimentación^(13)^.

En cuanto a la estimulación auditiva, la música suave ayudó a regular el sistema nervioso, aliviar la ansiedad, promover la secreción de jugos digestivos y favorecer la digestión y la absorción. El tono de voz suave del personal de enfermería también contribuyó a una experiencia de alimentación positiva, mejorando la cooperación y facilitando procesos de alimentación más fluidos. En cuanto a la estimulación olfativa, el aroma de la leche materna activaba el reflejo de búsqueda, aumentaba el deseo de alimentarse y mejoraba el éxito de la alimentación. Además, un protocolo de alimentación personalizado garantizaba que se satisfacían las necesidades nutricionales, mientras que una variedad de experiencias gustativas favorecía el desarrollo del sentido del gusto y mejoraba la aceptación de los alimentos^(14,15)^.

El estudio reveló que las puntuaciones NBNA y Apgar eran significativamente más altas en el grupo de intervención que en el grupo de control (P < 0,05), lo que indicaba una mejora en el desarrollo neurológico y fisiológico de los bebés prematuros. Las mejoras en la alimentación, impulsadas por el tacto sensorial multidimensional, aumentaron directamente la ingesta nutricional, lo que proporcionó una base sólida para el crecimiento y el desarrollo general. Además, un entorno sensorial cómodo y unos métodos de alimentación adecuados facilitaron la motilidad gastrointestinal y la secreción de jugos digestivos, mejorando la eficiencia de la digestión y la absorción de nutrientes. Estas mejoras fueron favorables para el aumento de peso y el desarrollo de diversos órganos y tejidos^(16,17)^.

Además, la estimulación sensorial diversa favoreció el desarrollo del sistema nervioso. La estimulación de los sentidos visual, auditivo y táctil enriqueció las conexiones sinápticas en el cerebro, promoviendo el desarrollo neurológico. Un sistema nervioso bien desarrollado ayudó a los bebés prematuros a controlar mejor los movimientos corporales, coordinar la deglución y la respiración, y mejorar aún más el crecimiento y el desarrollo^(18)^.

El estudio también demostró que un entorno sensorial confortable, combinado con la presencia de la familia y una atención de enfermería atenta, reducía las respuestas al estrés y estabilizaba la respiración de los bebés prematuros. Por ejemplo, la música suave y el contacto piel con piel ayudaron a aliviar la ansiedad y a prevenir la respiración irregular causada por las fluctuaciones emocionales. Una ingesta nutricional adecuada y una función circulatoria correcta garantizaban un buen suministro de sangre a la piel, lo que daba como resultado una tez sana y sonrosada. De este modo, un entorno sensorial favorable y unas intervenciones de enfermería eficaces mejoraban el estado de salud general y los signos físicos de los bebés prematuros^(19,20)^.

Aunque este estudio proporciona resultados sólidos y fiables, tiene varias limitaciones. El tamaño de la muestra era relativamente pequeño y todos los participantes fueron reclutados en un único centro de estudio, lo que puede introducir un sesgo de selección. Las investigaciones futuras deberían tener como objetivo ampliar el tamaño de la muestra e incluir una gama más amplia de indicadores de observación para validar y enriquecer los resultados actuales. Además, el uso de una muestra de conveniencia, basada en todas las admisiones elegibles durante el período de estudio, puede limitar la generalización de los resultados. Dado que los participantes no fueron seleccionados al azar de una población más amplia, no se puede descartar por completo la posibilidad de un sesgo de selección. Por lo tanto, se debe actuar con cautela al aplicar estos resultados a otras poblaciones o entornos clínicos.

## CONCLUSIÓN

En conclusión, el sistema de enfermería multidimensional sensorial táctil, acompañado por la familia, en todo el proceso de intervención alimentaria para bebés prematuros demuestra una eficacia significativa en la enfermería, al tiempo que mejora el crecimiento, el desarrollo y el proceso de alimentación.

## Data Availability

Todo el conjunto de datos que apoya los resultados de este estudo fue publicado en el próprio artículo.
